# Community health workers’ knowledge of Ubuntu informed care in tuberculosis, HIV, and AIDS in Gauteng province

**DOI:** 10.4102/curationis.v48i1.2679

**Published:** 2025-02-20

**Authors:** Rodwell Gundo, Nombulelo V. Sepeng, Robert Lavhelani, Mabitja Moeta, Maurine Musie, Raikane J. Seretlo, Fhumulani M. Mulaudzi

**Affiliations:** 1 Department of Nursing Science, Faculty of Health Sciences, University of Pretoria, Pretoria, South Africa; 2 Department of Public Health, Faculty of Health Sciences, Sefako Makgatho Health Sciences University, Pretoria, South Africa

**Keywords:** acquired immunodeficiency syndrome, HIV, South Africa, tuberculosis, surveys, questionnaires

## Abstract

**Background:**

Community health workers (CHWs) work with community members who experience various health problems. They assist community members to lead a healthy life and achieve an acceptable health status. To achieve this, there is a need for CHWs to apply Ubuntu philosophy when providing care related tuberculosis (TB), Human Immunodeficiency Virus (HIV), and acquired immunodeficiency syndrome (AIDS).

**Objectives:**

The aim of this study was to assess CHW’s knowledge of Ubuntu-informed Care in tuberculosis, HIV, and AIDS Services in Gauteng province, South Africa.

**Method:**

A total of 380 CHWs were recruited from a district in Gauteng province to participate in a training on the management of TB, HIV and AIDS. A questionnaire with 40 multiple choice questions was administered to the CHWs before the training. SPSS version 28 was used to analyse the data.

**Results:**

The scores ranged from 9 to 33 out of 40 (M = 21.6, s.d. = 4.2). Out of the 380 participants, 274 (72.1%) passed the pretest while 106 participants (27.9%) failed. The highest mean scores were achieved by female participants (M = 21.6, s.d. = 4.3), participants aged 21–30 years (M = 21.8, s.d. = 4.1) and participants with additional course qualification after Grade 12 (M = 23.5, s.d. = 3.4).

**Conclusion:**

The findings highlight the need for targeted training interventions to improve the knowledge of CHWs on TB, HIV and AIDS.

**Contribution:**

This study adds to the literature on the need for inclusion of Ubuntu when caring for people living with HIV and TB.

## Introduction

Tuberculosis (TB), Human Immunodeficiency Virus (HIV) and acquired immunodeficiency syndrome (AIDS) are public health issues globally. South Africa is one of the countries with high prevalence of TB, HIV and AIDS in the general population. The total number of people diagnosed with TB in the country accounted for 3.6% of the total cases globally in 2019, and those living with HIV (PLWHIV) were estimated to be 8.45 million in 2022 (Statistics South Africa 2022; World Health Organization [WHO] 2020). However, South Africa has made significant progress in turning the tide over the last decade by adopting WHO recommended diagnostic technologies and therapies for both prevention and management of the disease such as TB. In addition, HIV management and access to antiretroviral treatment (ART) have improved (Marinda et al. 2017; National Institute for Communicable Diseases 2022).

In 2021, 74% of people living with HIV were on ART in South Africa (Joint United Nations Programme on HIV/AIDS 2021). With increased access to ART, the number of AIDS-related deaths has declined from 278, 741 in 2007 to 85, 796 in 2022 (Statistics South Africa 2022). The programme is mainly managed by nurses who are responsible for dispensing the ART in primary healthcare settings while CHWs provide a supportive role (Mottiar & Lodge 2018). The CHWs are ancillary workers recruited in communities to provide promotive and preventive healthcare to the communities to reduce the burden placed on nurses in primary healthcare facilities (Knettel et al. 2021). A study by Knettel et al. (2021) conducted in Tanzania revealed that CHWs are well placed to establish rapport, trust, source of education and encouragement to clients. Among other duties, the CHWs assist in the prevention of TB, HIV, and AIDS and improve care across the continuum of care, on both individual and community levels (Datiko et al. 2015). The CHWs have helped to reduce maternal and child morbidity and mortality rates and the burden of communicable and non-communicable diseases in low- and middle-income countries (LMICs) (Feroz, Jabeen & Saleem 2015).

However, the CHWs are not given adequate support by health systems in the LMICs (Feroz et al. 2015). Despite the lack of support, their scope of practice has expanded in recent years because of changes in the healthcare system (Schleiff et al. 2021). In South Africa, some CHWs are appointed as primary healthcare ward-based teams and are paid stipends while others are still working on a voluntary basis. Their scope of practice ranges from pretreatment counselling, assisting with initiation and uptake of ART, TB screening, bed bath and feeding of those who are critically ill, providing health education, HIV testing and many other tasks that they provide based on circumstances of the patient during home visits. The WHO (2007) guidelines on task shifting suggested that the low categories must be trained before they are given tasks, which are beyond their scope of practice.

A study by Hughes et al. (2012) that was conducted in Free State revealed that more than 95% of CHWs were knowledgeable about TB and HIV, with only a few being unaware of important TB and HIV facts. This is despite not receiving any training related to basic HIV counselling and testing, as well as basic TB directly observed treatment support. Another study in the Eastern Cape found that a simple, user-friendly TB booklet with pictograms increased CHW knowledge. However, the country lacks standardised training manuals to provide CHWs with knowledge of tuberculosis, HIV, and AIDS (Okeyo & Dowse 2018). As a result, it is critical to assess CHWs’ knowledge of TB and HIV and AIDS in order to identify their learning needs, which will inform the development of a standardised training programme for CHWs to improve their competence in healthcare delivery.

Furthermore, Mwai et al. (2013) reported that CHWs improved the reach, uptake, and quality of HIV services, as well as the dignity, quality of life, and retention in care of HIV-positive people. While some participants believed that CHWs do an excellent job of maintaining confidentiality, others believed that CHWs can occasionally compromise confidentiality, limiting their ability to effectively facilitate linkage to care and management of chronic diseases (Rachlis et al. 2016).

The CHWs generally provide TB, HIV, and AIDS care in the communities from which they come, necessitating the use of Ubuntu when providing care to them. Ubuntu means ‘Ubuntu ngu muntu nga bantu’ in Xhosa, which translates to ‘person is a person through others’ (Moloko-Phiri et al. 2023; Mulaudzi et al. 2022; Mwai et al. 2013). Ubuntu is widely regarded by Africans as a form of human interconnectedness and dignity towards others, first in one’s cultural group and then to all other human beings (Waghid & Smeyers 2012). As a result, there is a need to educate CHWs on the importance of incorporating Ubuntu into their patient care, which promotes dignity towards others. Thus, leading to the prevention of disclosing confidential information of the patients. Furthermore, Ubuntu emphasises the importance of respecting and valuing others, as well as compassion and care for one another. Despite this, there is little documented evidence of community health workers’ knowledge of Ubuntu-Informed Care in TB, HIV, and AIDS services in Gauteng province, South Africa.

### Aim

The study aimed to assess CHW’s knowledge of Ubuntu-Informed Care in TB, HIV, and AIDS Services in Gauteng province, South Africa.

### Objectives

The study’s objectives were as follows: (1) to assess CHWs’ knowledge of TB, HIV and AIDS in a selected district in Gauteng province of South Africa; and, (2) to assess CHW’s knowledge of Ubuntu-Informed Care in TB, HIV, and AIDS Services in Gauteng province, South Africa.

## Research methods and design

### Design

A cross-sectional survey was used to assess the CHWs knowledge of Ubuntu-Informed Care in TB, HIV, and AIDS Services in Gauteng province, South Africa at the beginning of a training.

### Study setting and participants

The training was conducted at a neutral venue in three townships of Gauteng province in South Africa. The province is an economic hub, which is densely populated with a population of 13.4 million (Statistics South Africa 2023). The public health system consists of central hospitals, regional tertiary hospitals and district hospitals. The primary healthcare (PHC) comprises community health centres and PHC clinics that provide ambulatory care services (White, Blaauw & Rispel 2020). According to Kim et al. (2021) the province has the largest population of people living with HIV (PLHIV), with a density of 13 862 women and 15 484 men per 5 km^2^. Regarding tuberculosis, the province has the highest prevalence such that people residing in the province are 4794 times more likely to develop TB compared to those who reside in Limpopo province (Maja & Maposa 2022). The participants in the survey were CHWs in three townships of Gauteng province in South Africa.

### Data collection tool

We developed a structured pretest with 40 multiple-choice questions based on modules of a training manual on the management of TB, HIV and AIDS, and the African philosophy of Ubuntu. Out of the 40 questions, 36 questions (1–31 and 36–40) were related to the management of TB, HIV and AIDS while four questions (32–35) assessed the participants knowledge about Ubuntu. The manual has 10 modules, and each module includes sections that focus on Ubuntu-informed care, which promotes respect, empathy, and interconnection in the context of HIV and TB services. The modules are as follows: Module 1 – Introduction to HIV and AIDS, Module 2 – Knowledge of sexually transmitted infections, HIV and AIDS, Module 3 – HIV counselling and testing, Module 4 – Treatment and support for people living with HIV and AIDS, Module 5 – Stigma, discrimination and ethical issues related to people living with HIV and AIDS, Module 6 – Overview of tuberculosis, Module 7 – Tuberculosis screening, testing and treatment, Module 8 – Advocacy, Module 9 – Tuberculosis and HIV co-infection, and Module 10 – Role of community healthcare providers. The training was piloted with 30 CHWs in the same province in December 2022. At the end of the pilot training, the participants recommended the use of a pretest to assess their knowledge on the management of TB, HIV and AIDS. Participants who attended the pilot training were not invited to attend the subsequent training sessions.

### Data collection process

The pretest was administered to 380 CHWs who accepted to attend a 5-day training on the management of TB, HIV and AIDS between January and April 2023. In each township, the facilitators of the training identified a neural venue for the training in consultation with coordinators of the CHWs. The test was administered to the participants on the first day of the training before the first lesson. For each question, the participants were requested to choose and circle the correct response out of the available options. To pass the test, each participant was expected to get a minimum total score of 20 out of 40. The completion of the test took 30–60 min.

### Data analysis

Data were entered into Microsoft Excel and later imported into SPSS version 28 for analysis. Descriptive statistics namely mean, standard deviation and frequencies were used to summarise the participants’ demographic characteristics and scores on the test. Inferential statistics such as independent samples *t*-test and one-way between-groups analysis of variance were used to compare the scores among different groups of the participants. Statistical significance was set at 0.05. The results are presented in tables and figures.

### Ethical considerations

The study was approved by the Research Ethics Committee of the Faculty of Health Sciences at the University of Pretoria, approval number 465/2020. The participants were provided with an information sheet about the training and those who accepted to attend the training provided a written consent. Code numbers were used to ensure anonymity of the participants.

## Results

### Demographic profile

Most of the participants were females, *n* = 290 (76.3%) while males were 88 (23.2%). Two participants (0.5%) did not indicate their gender. The age ranged from 19 to 57 (M = 31.9, s.d. = 6.8) with the majority aged between 21 and 40 years as presented in Table 1.

**TABLE 1:** Demographic profile of the participants (*N* = 380).

Variable	*n*	%
**Training site**		
Hatfield	164	43.2
Mamelodi	128	33.7
Soshanguve	88	23.2
**Age group (years)**		
11–20	4	1.1
21–30	163	42.9
31–40	174	45.8
41–50	32	8.4
51–60	5	1.3
Not indicated	2	0.5
**Level of education**		
Below Grade 12	39	10.3
Grade 12	249	65.5
Courses after Grade 12	53	13.9
Degree	16	4.2
Not indicated	23	6.1

### Performance of the participants

Out of the 380 participants, 274 (72.1%) passed the pre-test while 106 participants (27.9%) failed. The scores ranged from 9 to 33 out of 40 (M = 21.58, s.d. = 4.24). Most participants passed question number 2 (85.5%, *n* = 325), 17 (84.5%, *n* = 321), 22 (86.8%, *n* = 330), 30 (84.2%, *n* = 320) and 37 (87.1%, *n* = 331). On the other hand, most participants failed questions 7 (80.8%, *n* = 307), 11 (76.8%, *n* = 292), 12 (73.7, *n* = 270), 13 (90%, *n* = 342), 18 (77.1%, *n* = 292), 23 (88.4%, *n* = 336), 24 (90%, *n* = 342) and 26 (70.5%, *n* = 268). The questions that yielded lowest scores were related to TB and prevention of mother-to-child transmission of HIV. The participants’ mean scores on questions specific to Ubuntu philosophy ranged from 0.63 (s.d. = 0.48) to 0.84 (s.d. = 0.37). The number of participants who passed the questions was as follows: question 32 (83.7%, *n* = 318), question 33 (62.9%, *n* = 239), question 34 (68.7%, *n* = 261) and question 35 (79.5%, *n* = 302). A summary of the scores is presented in Table 2. Out of the 380 participants, 274 (72.1%) passed the pre-test while 106 participants (27.9%) failed. The scores ranged from 9 to 33 out of 40 (M = 21.58, s.d. = 4.24). Most participants passed question number 2 (85.5%, *n* = 325), 17 (84.5%, *n* = 321), 22 (86.8%, *n* = 330), 30 (84.2%, *n* = 320) and 37 (87.1%, *n* = 331). On the other hand, most participants failed questions 7 (80.8%, *n* = 307), 11 (76.8%, *n* = 292), 12 (73.7, *n* = 270), 13 (90%, *n* = 342), 18 (77.1%, *n* = 292), 23 (88.4%, *n* = 336), 24 (90%, *n* = 342) and 26 (70.5%, *n* = 268). The questions that yielded lowest scores were related to TB and prevention of mother-to-child transmission of HIV. The participants’ mean scores on questions specific to Ubuntu philosophy ranged from 0.63 (s.d. = 0.48) to 0.84 (s.d. = 0.37). The number of participants who passed the questions was as follows: question 32 (83.7%, *n* = 318), question 33 (62.9%, *n* = 239), question 34 (68.7%, *n* = 261) and question 35 (79.5%, *n* = 302). A summary of the scores is presented in Table 2.

**TABLE 2:** Questions with highest and lowest mean scores.

Number	Question	Mean	s.d.
2	Individuals cannot become infected with HIV through ordinary day-to-day contact	0.84	0.37
7	The interventions for the prevention of mother-to-child transmission of HIV include the following except	0.2	0.4†
11	The tests or investigations that are used to detect tuberculosis (TB) bacteria include the following except one of the answers	0.23	0.42†
12	The advantages of using a Direct Observation Treatment (DOT) supporter in the treatment of tuberculosis include the following except	0.26	0.44†
13	The following is a side effect of TB drugs	0.1	0.3†
17	What is TB and HIV co-infection?	0.84	0.37
18	Which tests can be performed to diagnose TB and HIV co-infection?	0.22	0.41†
22	Which of the following poses a risk to transmission of TB (tuberculosis)?	0.87	0.34
23	The most common signs and symptoms of extra-pulmonary TB disease identified during TB screening is	0.11	0.32†
24	The most reliable TB test used to detect TB bacteria is	0.1	0.3†
26	What is the type of physical activity that may be used by healthcare providers caring for HIV and TB patients to enhance their coping mechanisms when experiencing stress?	0.29	0.46†
30	Which one of the following actions represents a proper approach to advocating for community resources?	0.84	0.37
31	Social groups are important for the following reasons except one of the answers	0.43	0.49
32	Ubuntu is an African philosophy which means:	0.84	0.37
33	Which of the following is a combination of the values of Ubuntu?	0.63	0.48
34	Which of the following Ubuntu values is reflected by Nurse B in the scenario?	0.69	0.46
35	Which of the following Ubuntu values would you refer to in the discussions?	0.79	0.40
36	A counsellor must disclose (share information) the status to the partner after counselling?	0.72	0.45
37	HIV testing and counselling should consider the readiness of the client to test	0.88	0.33
38	A counsellor must disclose (share information) the status to the partner after counselling	0.59	0.49
39	An HIV-positive result does not have to be repeated immediately	0.45	0.49
40	One method of testing for HIV is antibody test	0.44	0.49

s.d., standard deviation.

† , Questions with low mean scores.

### Comparison of the overall mean scores among different groups

#### Gender

**FIGURE 1:**
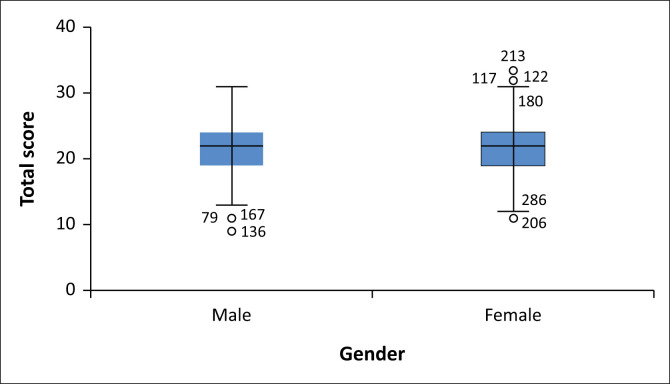
The distribution of the total scores by gender.

Female participants achieved a higher mean score (M = 21.64, s.d. = 4.31) compared to male participants (M = 21.35, s.d. = 4.09); however, statistical analysis revealed that this difference was not significant (*p* = 0.57). The magnitude of the differences in the means (mean difference = -0.29, 95% CI: -1.31 to 0.73). The results are summarised in Figure 1.

#### Age

Participants were divided into five groups according to their age (Group 1: 11–20 years; Group 2: 21–30 years; Group 3: 31–40 years; Group 4: 41–50 years; and Group 5: 51–60 years). The highest mean score was recorded among participants aged 21–30 years (M = 21.83, s.d. = 4.1), while the 51–60 age group achieved the lowest mean score (M = 18.40, s.d. = 5.51). There was no statistically significant difference at the *p* < 0.05 in the total scores for the five age groups: F (4, 373) = 1.41, *p* = 0.23.

#### Level of education

**FIGURE 2:**
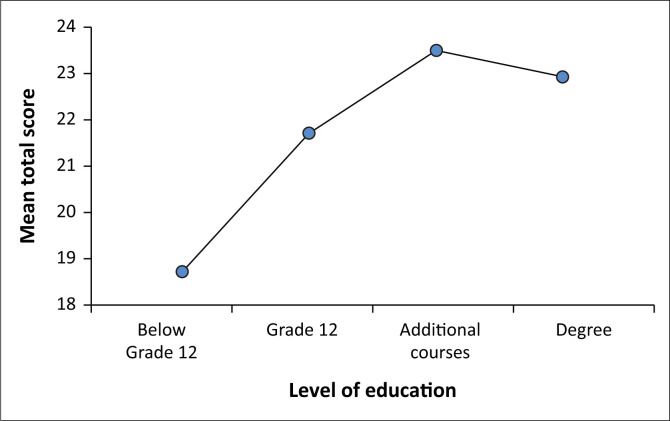
The distribution of the mean scores by level of education.

Participants were divided into four groups according to their qualification (Group 1: Below Grade 12; Group 2: Grade 12; Group 3: Additional course after Grade 12; and Group 4: Degree). Participants with additional course qualification after Grade 12 achieved a high mean score, 23.51 (s.d. = 3.38) while participants whose qualification was below Grade 12 had the lowest mean score, 18.72 (s.d. = 3.53). A summary of the results is presented in Figure 2. There was a statistically significant difference in total scores for the four groups: F (3, 353) = 11.08, *p* = 0.00. Post hoc comparisons using the Tukey Honest Significant Difference (HSD) test indicated that the mean score for Group 1 (M = 18.72, s.d. = 3.53) was significantly different from Group 2 (M = 21.70, s.d. = 4.19), Group 3 (M = 23.51, s.d. = 3.38) and Group 4 (M = 22.94, s.d. = 4.99). Similarly, the mean score for Group 2 (M = 21.70, s.d. = 4.19) was significantly different from Group 3 (M = 23.51, s.d. = 3.38), *p* = 0.02.

## Discussion

The aim of this study was to assess CHW’s knowledge of Ubuntu-Informed Care in TB, HIV, and AIDS Services in Gauteng province, South Africa. Most participants passed the test. However, the mean score was slightly above the pass mark, which demonstrates that the participants had limited understanding of TB, HIV and AIDS. Most participants failed questions related to TB and prevention of mother-to-child transmission of HIV. However, most participants correctly answered the questions specific to Ubuntu philosophy. With regard to demographic characteristics, level of education had an impact on the total score such that participants whose level of education was below Grade 12 had the lowest scores.

Our results are similar to previous studies Minnery et al. (2013); Rachlis et al. (2016). A study by Minnery et al. (2013) which was conducted in Peru reported areas of concern regarding the knowledge of CHWs on the diagnosis and management of TB, HIV, and AIDS. Similarly, the CHWs’ lack of knowledge on HIV treatment and management was reported by Rachlis et al. (2016) in a study that was conducted in Kenya. In addition, similar studies showed that CHWs often do not have proper training in HIV management, limiting their capacity to deliver effective treatment (Ngcobo et al. 2022; Rajabiun et al. 2021). These findings are worrisome especially in low- and middle-income countries such as South Africa where HIV, AIDS, and TB exert a heavy toll. There is a need to empower the CHWs and accelerate efforts in the fight against TB, HIV and AIDS to achieve the Sustainable Developments Goals number 3, which ‘aspires to ensure health and well-being for all, including a commitment to end the epidemics of HIV/AIDS, tuberculosis, Malaria and other communicable diseases by 2030’ (United Nations Development Programme 2023).

The CHWs are key players in the fight against HIV infection, prevention, and management of HIV and AIDS in resource-restricted communities (Cianelli et al. 2011). They are the first point of contact with patients in the communities and are often responsible for providing health education and other important information to patients regarding health-related matters (Okeyo & Dowse 2016). The perception that communities have about CHWs depends on how well they can communicate health information pertaining to diseases such as TB, HIV, and AIDS. Therefore, it is imperative that they possess accurate knowledge of health problems that are prevalent in their communities. The results of this study support calls for the need to use different strategies to empower the CHWs. The suggested strategies include training of the CHWs to ensure efficient timely care and management of TB, HIV, and AIDS. (Rachlis et al. 2016; Sander et al. 2015). With proper education and training, the CHWs can assess various illness and make necessary follow-up in the communities (Rachlis et al. 2016).

In addition, our results showed that CHWs with low level of education achieved low scores. In the previously cited study by Rachlis et al. (2016) the lack of knowledge among CHWs was also attributed to low level of education or a lack of formalised training for the CHWs. An implication of this result is the need to seriously consider level of education when recruiting the CHWs and the need for supportive supervision of the CHWs to introduce and practice Ubuntu-informed care. The findings also support suggestions that CHWs should be provided with resources such as posters, charts, pamphlets, and brochures for health education in the community (Mukanga et al. 2011; Rachlis et al. 2016). The CHW can also give out these resources in the community to disseminate information about different diseases. The study’s findings revealed that CHWs had a good understanding of how the Ubuntu philosophy and values could be applied when testing and managing clients with tuberculosis, HIV, and AIDS. According to a previous study, there is a need to promote the use of Ubuntu concepts in improving safe sexual practices and positive attitudes towards PLHIV in Africa (Knettel et al. 2021). Our study supports Tarkang, Pencille and Komesuor (2018) findings that Ubuntu encourages values of inclusiveness and respect, which can help reduce stigma against PLHIV, and emphasises that by cultivating a culture of empathy and compassion, Ubuntu may result in better sexual health outcomes, especially among youth. Although most of the CHWs answered the questions on Ubuntu philosophy correctly, our interactions during training revealed limitations in their overall knowledge, emphasising the significance of including these ideas into training programmes. This concept can be a valuable resource for maintaining confidentiality, which is a critical requirement when delivering HIV and AIDS health promotion interventions.

### Strengths

One of the strengths of our study is the large sample of CHWs. This is also the first study to assess the CHW’s knowledge on Ubuntu-informed care regarding TB, HIV and AIDS. In addition, the questionnaire that we used had not been used before in any of the previous studies. Despite this limitation, the involvement of local experts in the development of the questionnaire helped to ensure that the items included in the questionnaire are relevant to the CHWs in South Africa.

### Limitations

This study’s reported findings were conducted in a single province, which limits their generalisability to other provinces. Furthermore, the pre-test training was given to groups of people with varying levels of education, which may have contributed to a lack of knowledge about HIV and AIDS prevention in maternal and child health.

## Conclusion and recommendations

The aim of this study was to assess the knowledge of CHWs on TB, HIV, and AIDS in a selected district of Gauteng province in South Africa. The results show that CHWs have limited knowledge on the management of TB, HIV and AIDS prevention in maternal and child healthcare. This highlights the need for targeted training interventions to improve the knowledge of CHWs on TB, HIV and AIDS in this area. Addressing the identified knowledge gaps is crucial to enhance the effectiveness of CHWs in community health promotion and disease prevention. Such interventions may have the potential to strengthen the healthcare system’s response to the challenges posed by TB, HIV and AIDS, ultimately contributing to improved health outcomes in the community. A further qualitative study could be conducted to explore the CHWs’ learning needs to inform interventions that are aimed at empowering them. Interventions that address the identified knowledge gaps can enhance the effectiveness of the CHWs and strengthen the healthcare system’s response to the challenges posed by TB, HIV and AIDS.
